# Helicobacter pylori infection, dementia and primary open-angle glaucoma: are they connected?

**DOI:** 10.1186/s12886-015-0006-2

**Published:** 2015-03-11

**Authors:** Fani Tsolaki, Jannis Kountouras, Fotios Topouzis, Magda Tsolaki

**Affiliations:** Ophthalmology Department, Hippokrateion General Hospital, Thessaloniki, Greece; Second Medical Department, Hippokrateion General Hospital, Aristotle University of Thessaloniki, Thessaloniki, Greece; Ophthalmology Department, Aristotle University of Thessaloniki, AHEPA Hospital, Thessaloniki, Greece; 3d Department of Neurology, Aristotle University of Thessaloniki, Papanikolaou General Hospital, Thessaloniki, Greece

**Keywords:** Dementia, Primary open-angle glaucoma, Helicobacter pylori, Neurodegenerative disease

## Abstract

**Background:**

The study aims to elucidate the putative association between various forms of dementia, including Alzheimer’s disease, primary open-angle glaucoma and Helicobacter pylori (*H.pylori*) infection in all possible combinations.

**Methods:**

We prospectively recruited for the study 156 patients, divided into a dementia group, a glaucoma group and two control groups. All patients were submitted to neuropsychological evaluation aiming to detect dementia, ophthalmological examination, aiming to detect glaucoma, and *H.pylori* diagnostic testing. The provided data were analyzed with the question of possible correlation between the aforementioned entities.

**Results:**

Positive correlations were found between Hp infection and dementia, Hp infection and glaucoma, as well as between dementia and glaucoma.

**Conclusions:**

The study confirmed the hypothesis that neurodegenerative diseases such as dementia and glaucoma are linked to each other and to *H.pylori* infection.

## Background

Alzheimer’s disease (AD), the main cause of dementia, affects more than 30 million people worldwide, with a rising tendency towards the number of 100 million patients estimated to suffer from the disease in the year 2050 [1,2]. Primary open angle glaucoma (POAG) is the second cause of blindness worldwide [3]. *H. pylori* is a common, Gram-negative bacterium found in the stomach. It has been shown to elicit an inflammatory response (gastritis) by altering stomach pH, and by secreting proteases and VacA. It has also been implicated in the development of gastroduodenal ulcer, gastric cancer, MALT-lymphoma and colorectal cancer [4-8].

Neurodegenerative disorders are believed to be linked to each other as well as to *H.pylori* infection in terms of epidemiology and etiology [9-13]. However, various studies investigating the issue have concluded to contradictory results [14-16]. For this reason, aim of the current prospective study is to elucidate the putative association between Alzheimer’s disease and other dementias (frontotemporal- dementia- FTD), dementia with Parkinson’s disease-PD, Lewy body dementia-LB), primary open-angle glaucoma (POAG) and *H.pylori* infection in all possible combinations, based on a wide range of diagnostic procedures, that would set the diagnosis of each disorder with accuracy and beyond doubt.

## Methods

The study was performed in compliance with the Helsinki Declaration and after the approval of the Ethics Committee of the Aristotle University of Thessaloniki. The patients, whose informed consent was obtained prior to the initiation of the study were divided in the following groups:

i. A group of patients with dementia (60 patients), which included patients with official diagnosis of AD (36 patients), PD (9 patients), LB (9 patients), FTD (6 patients) [17-20].
ii. A group of patients with POAG (35 patients).
iii. A control group of 31 subjects found negative for dementia and glaucoma. They were used for the analysis of Hp infection incidence in patients with dementia and glaucoma.
iv. A second control group of 30 subjects recruited from an emergency ophthalmology department, used for the analysis of correlation between dementia and glaucoma.

### Neuropsychological battery

All participants were checked for dementia with the application of a neuropsychological battery consisting of the Mini Mental State Examination, the Functional Rating Scale for Symptoms of Delirium, the Neuropsychiatric Inventory, the Hindi Mental State Examination and the Geriatric Depression Scale [21-28].

### Ophthalmological examination

The ocular examination was based on visual acquity control (reading of an optotype from a distance of 5 meters), tonometry with a Goldmann’s applanation tonometer [29] after application of a local anesthetic (hydrochloric proxymetacaine 0.5%), and fundoscopy after the application of tropicamide solution 0.5% and of the a-adrenergic stimulator phenylefrine in a solution of 5%. The examination was concluded with a control of visual fields in a static perimetry apparatus type Octopus 900 (Haag-Streit, Switzerland). Diagnostic criteria for glaucoma were the ones provided by the Thessaloniki Eye Study [30].

### Helicobacter pylori infection diagnosis

The examination for the Hp infection was based on gastroscopy and histological examination of the retrieved tissue specimens. Gastroscopy was performed after induction of sedation with midazolame (Dormicum) in an iv. administration and in a dosage of 3–5 mg, according to the patient’s weight. This was followed by application of xylocaine to the nasopharynx in a spray form. The endoscopic instrument that was used for the research was an Olympus gastroscope.

Histological examination of the tissue specimens was based on a Cresyl-Violet staining. In a small number of patients (5), who couldn’t be submitted to endoscopy due to lack of cooperation, we performed a serological examination of IgG antibodies against *H.pylori*.

### Statistical analysis

The statistical analysis was based for the comparison of percentages on the *x*^2^ criterion. For the quantitative parameters the analysis was based on the non parametric Mann–Whitney-Wilcoxon test. The quantitative parameters were expressed as mean ± standard deviation. The levels for statistical significance were set for values of p < 0.05. The program we used for the statistical analysis was the SPSS 21.0 v.

## Results and discussion

The study concluded to positive correlations between HP infection and dementia and *H.pylori* infection and glaucoma. This was expressed as an augmented frequency of *H.pylori* infection in the two groups in comparison to the frequency of the control group (68.33% in patients with dementia vs 45.16%, p < 0.05, 68.57% in patients with glaucoma vs 45.16%, p < 0.05). It has also shown a positive correlation between dementia and glaucoma, both in the form of augmented frequency of glaucoma in patients with AD and PD (16.66% vs 0%,) as well as in the form of augmented frequency of dementia (AD and FTD) in patients with glaucoma (16.66% vs 0%), in comparison to the frequency in the control group. The aforementioned findings are depicted in Tables 1, 2, 3, 4 and 5.

**Table 1:** **Comparative data regarding age and gender between patients with dementia, glaucoma and the subjects of the 2 control groups**

	**Patients with dementia n = 60**	**Patients with glaucoma n = 35**	**First control group n = 31**	**Second control group (n = 30)**	**Statistical significance**
**Age**	61.34 ± 6.526	62.18 ± 5.04	62.41 ± 4.49	61.48 ± 2.8	*p* > 0,05
**Male patients**	28 (46.66%)	14 (46.66%)	14 (45.16%)	13 (43.44%)	*p* > 0,05

The table shows comparative data in regard to age and gender between the patients with dementia, glaucoma and the patients of the two control groups. The comparison was performed with the non parametric Mann–Whitney-Wilcoxon test for the age analysis and the *x*2 for the gender distribution. The level of statistical significance was set at a threshold for p < 0.05.

**Table 2:** **Helicobacter pylori infection in patients with dementia and control patients**

**Dementia**	**Percentage in %**	**Control group**	**Percentage in %**	**Statistical significance**
41/60	68.33	14/31	45.16	*p* < 0.05
**Alzheimer’s disease**	69.44	14/31	45.16	*p* < 0.05
25/36				
**Parkinson’s dementia**	66.67	14/31	45.16	*p* < 0.05
6/9				
**Frontotemporal dementia**	66.67	15/31	45.16	*p* < 0.05
4/6				
**Lewy body dementia**	66.67	15/31	45.16	*p* < 0.05
6/9				

The table depicts comparative data between patients with dementia in total and per diagnosis and members of the control group in regard to Hp infection. For the statistical analysis the *x*2 criterion was applied. The level of statistical significance was set at a threshold for p < 0.05.

**Table 3:** **Hp infection in glaucoma and control group patients**

**Patients with glaucoma**	**Percentage in %**	**Control group**	**Percentage in %**	**Statistical significance**
**24/35**	**68.57%**	**14/31**	**45.16**	***p*** **< 0.05**

The table shows comparative data between patients with glaucoma and subjects of the control group in regard to Hp infection. For the statistical analysis the *x*2 criterion was applied. The level of statistical significance was set at a value for p < 0.05.

**Table 4:** **Frequency of glaucoma in patients with dementia – frequency of glaucoma in patients of the control group**

**Patients with dementia**	**Percentage in %**	**Control group**	**Percentage in %**	**Statistical significance**
10/60	16.66	The control group had no members with glaucoma	The control group had no members with glaucoma	p < 0.01
**Patients with Alzheimer’s disease**	19.44			
7/36				
**Patients with Parkinson’s dementia**	33.33			
3/9				

The table shows comparative data regarding the frequency of glaucoma in patients with dementia and control group. The control group had no members with glaucoma. For the statistical analysis the *x*2 criterion was applied. The level of statistical significance was set at a value for p < 0.05.

**Table 5:** **Frequency of dementia in patients with glaucoma and control group**

**Patients with glaucoma**	**Percentage in %**	**Control group**	**Percentage in %**	**Statistical significance**
5/35 (4 patients with Alzheimer’s disease and 1 with Frontotemporal Dementia)	14.28	The control group members had no dementia		

The table shows comparative data regarding the frequency of dementia in patients with glaucoma and control patients. The control group members had no dementia. For the statistical analysis the *x*2 criterion was applied. The level of statistical significance was set at a value for p < 0.05.

The positive findings of the study in regard to the association of dementia (particularly AD) and Hp infection, POAG and *H.pylori* infection and finally, between dementia and POAG come to confirm the related conclusions reached by Kountouras et al. [9,11,12] and Bayer et al. [13].

Which common pathophysiological pathways stand behind the examined entities is not yet known. A possible explanation is that *H.pylori* infection initiates mechanisms of humoral and cellular immune response, which create crossreactions due to common genetic components with the ones found in nerve tissue; what follows are apoptosis-related mechanisms of cell destruction which are frequent in neurodegenerative diseases, such as dementia and glaucoma [31].

Another theory that might explain the findings of the study is that of the augmented permeability of the blood–brain barrier in patients with neurodegenerative diseases, such as multiple sclerosis [32,33]; this phenomenon may give to the *H.pylori* the chance to enter the barrier and accelerate or influence their course. Similarly, Deretzi et al. have recently [34] proposed a theory that *H.pylori* or other pathogens may follow a distinct pathway to bypass the blood–brain barrier and cause damage to the brain. More specifically, they use the gastrointestinal tract (GIT) retrograde axonal transport through sensory or motor fibres in order to invade the central nervous system.

The findings of the study may have important practical applications for our patients in the future. They may lead to a widely accepted eradication of *H.pylori* as a measure for prevention against dementia and glaucoma. They also may lead to the establishment of routine diagnostic examinations for glaucoma in patients with dementia and of dementia in patients with glaucoma, which would influence the quality of life of these patients positively. Continued research in this field in the epidemiological, but more intensely in the molecular level, might further clarify common biochemical pathways linking dementia, glaucoma and Hp infection.

## Conclusions

i. *H.pylori* infection is more frequent in patients suffering from AD and other forms of dementia than in the general population.
ii. *H.pylori* infection is more frequent in patients suffering from POAG than in the general population.
iii. POAG is more frequent in patients suffering from dementia (AD and PD) than in the general population.
iv. Dementia (AD and FTD) is more frequent in patients with POAG than in the general population.

## Abbreviations

AD: Αlzheimer’s disease
POAG: Primary open-angle glaucoma
Hp: Helicobacter pylori
PD: Dementia with Parkinson’s disease
FTD: Frontotemporal dementia
LB: Lewy body dementia
